# Different cellular effects of four anti-inflammatory eye drops on human corneal epithelial cells: independent in active components

**Published:** 2011-12-06

**Authors:** Mingli Qu, Yao Wang, Lingling Yang, Qingjun Zhou

**Affiliations:** State Key Laboratory Cultivation Base, Shandong Provincial Key Laboratory of Ophthalmology, Shandong Eye Institute, Shandong Academy of medical Sciences, Qingdao, China

## Abstract

**Purpose:**

To evaluate and compare the cellular effects of four commercially available anti-inflammatory eye drops and their active components on human corneal epithelial cells (HCECs) in vitro.

**Methods:**

The cellular effects of four eye drops (Bromfenac Sodium Hydrate Eye Drops, Pranoprofen Eye Drops, Diclofenac Sodium Eye Drops, and Tobramycin & Dex Eye Drops) and their corresponding active components were evaluated in an HCEC line with five in vitro assays. Cell proliferation and migration were measured using 3-(4,5)-dimethylthiahiazo (-z-y1)-3 5-di-phenytetrazoliumromide (MTT) assay and transwell migration assay. Cell damage was determined with the lactate dehydrogenase (LDH) assay. Cell viability and median lethal time (LT_50_) were measured by 7-amino-actinomycin D (7-AAD) staining and flow cytometry analysis.

**Results:**

Cellular effects after exposure of HCECs to the four anti-inflammatory eye drops were concentration dependent. The differences of cellular toxicity on cell proliferation became significant at lower concentrations (<0.002%). Diclofenac Sodium Eye Drops showed significant increasing effects on cell damage and viability when compared with the other three solutions. Tobramycin & Dex Eye Drops inhibited the migration of HCECs significantly. Tobramycin & Dex Eye Drops showed the quickest effect on cell viability: the LT_50_ was 3.28, 9.23, 10.38, and 23.80 min for Tobramycin & Dex Eye Drops, Diclofenac Sodium Eye Drops, Pranoprofen Eye Drops, and Bromfenac Sodium Hydrate Eye Drops, respectively. However, the comparisons of cellular toxicity revealed significant differences between the eye drops and their active components under the same concentration. The corneal epithelial toxicity differences among the active components of the four eye drops became significant as higher concentration (>0.020%).

**Conclusions:**

The four anti-inflammatory eye drops showed different cellular effects on HCECs, and the toxicity was not related with their active components, which provides new reference for the clinical application and drug research and development.

## Introduction

Anti-inflammatory agents have been widely used systemically and topically in the treatment of ocular inflammatory conditions following cataract surgery and glaucoma surgery [1]. Ophthalmic corticosteroids have been the first-line therapy to reduce the postoperative inflammation by inhibition of phospholipase A2 production. However, topical corticosteroid application may lead to impaired corneal wound healing, cataract formation, glaucoma by elevated intraocular pressure (IOP), and increased risk of infection [2]. Non-steroidal anti-inflammatory drugs (NSAIDs) exert an equivalent anti-inflammatory effect by inhibiting cyclooxygenases (COXs) that mediate the breakdown of arachidonic acid to produce prostaglandins and other metabolic products in the arachidonic acid cascade, and diclofenac sodium can also interfere with the lipoxygenase pathway [3]. Moreover, these drugs have shown an increasing use in managing posttraumatic or postoperative pain because of their analgesic effects, and they treat allergic ocular disorders by inhibiting prostaglandin synthesis [4,5]. Compared with corticosteroids, NSAIDs have become widely accepted to control ocular inflammation and pain because they do not raise IOP and they reduce the risk of secondary cataracts or infections [6].

Although the complications of NSAIDs are considerably less frequent than those of corticosteroids, some reports describe the adverse effects from topical NSAIDs, especially on conditions such as autoimmune disease or dry eyes. The most commonly reported adverse effects from topical NSAIDs include stinging, irritation, superficial punctate keratitis, corneal infiltrates, and melting [7]. In addition, NSAIDs inhibit COX activity in the arachidonic acid cascade and decrease prostaglandin synthesis. Prostaglandins are essential for protein and DNA synthesis in the epidermal cells; therefore, postoperative use of NSAIDs (especially diclofenac sodium) may affect corneal epithelial wound healing [3,8]. As reported previously, diclofenac sodium significantly delays early wound healing in the scraped rabbit corneal epithelium and re-epithelialization after penetrating keratoplasty [8].

Although a few articles have compared the effects of these anti-inflammatory eye drops on the proliferation or migration of corneal epithelial cells or conjunctival epithelial cells [9], little is known about the acute or early corneal cytotoxicity and the toxicity of their active components. In the present study, we systemically compared the cellular effects of three NSAIDs (Bromfenac Sodium Hydrate Eye Drops, Pranoprofen Eye Drops, and Diclofenac Sodium Eye Drops) and one corticosteroid solution (Tobramycin & Dex Eye Drops) and their corresponding active components on immortalized human corneal epithelial cells (HCECs) by using five in vitro assays, including 3-(4,5)-dimethylthiahiazo (-z-y1)-3 5-di-phenytetrazoliumromide (MTT) assay, transwell migration assay, lactate dehydrogenase (LDH) assay, 7-amino-actinomycin D (7-AAD) staining and flow cytometry analysis. We found that the four anti-inflammatory eye drops showed different cellular effects on HCECs, and the toxicity was not related with their active components, which provides new reference for the clinical application and drug research and development.

## Methods

### Eye drops

Senju Pharmaceutical Co., Ltd. (Osaka, Japan) provided the four anti-inflammatory eye drops and their active components for this study, including three NSAIDs (Bromfenac Sodium Hydrate Eye Drops, Pranoprofen Eye Drops, and Diclofenac Sodium Eye Drops) and one corticosteroid (Tobramycin & Dex Eye Drops). The active components of the four eye drops were dissolved in DMEM/F-12 (Invitrogen, Carlsbad, CA) media as the same concentration as that of the commercial solutions. Table 1 lists the main information of the four anti-inflammatory eye drops.

**Table 1:** Anti-inflammatory eye drops used in the present study.

**Generic name**	**Active component**	**Preservative**	**pH**
Bromfenac Sodium Hydrate Eye Drops (Senju, Japan)	Bromfenac sodium hydrate (0.1%)	0.005% BAC*	7.5–8.5
Pranoprofen Eye Drops (Senju, Japan)	Pranoprofen (0.1%)	0.007% BAC*	7.5–8.5
Diclofenac Sodium Eye Drops (Sinqi, China)	Diclofenac sodium (0.1%)	NK†	7.0–8.5
Tobramycin & Dex Eye Drops (Alcon, USA)	Tobramycin (0.3%) & Dex (0.1%)	0.001% BAC*	5.3‡

*BAC: Benzalkonium chloride; †NK: not known; ‡: measured result.

### Human corneal epithelial cell culture

Simian virus 40–immortalized HCECs were provided by Choun-Ki Joo (The Catholic University of Korea, School of Medicine, Seoul, Korea). The cells were cultured in DMEM/F-12 (1:1) media, 5% fetal bovine serum (FBS; Gibco-BRL, Grand Island, NY), 5 μg/ml insulin (Sigma, St. Louis, MO), 0.1 ng/ml cholera toxin (EMD Biosciences, San Diego, CA), 10 ng/ml human epidermal growth factor (hEGF; R&D Systems, Minneapolis, MN), and 0.5% dimethyl sulfoxide (DMSO; Sigma) in a humidified 5% CO_2_ incubator at 37 °C [10].

### Cell proliferation assay

Cell proliferation was measured using MTT assay. Briefly, HCECs were inoculated 3,000 cells per well in 96-well plates and allowed to adhere. The media were aspirated and replaced with the media containing drugs with different dilutions for 24 h, followed by 4-h incubation with MTT. The MTT transformed crystals were dissolved in DMSO, and absorbance at 490 nm was measured using a microplate reader (SpectraMax M2, Molecular Devices, Menlo Park, CA).

### Cell migration assay

Migration assay was performed on a 6.5-mm-diameter transwell chamber with an 8-μm pore size (Corning Costar, Cambridge, MA). HCECs (5×10^4^ cells) in the serum-free DMEM/F-12 media were inoculated on the top of the transwell chamber and allowed to migrate toward DMEM/F-12 medium containing 1% FBS supplemented with different drugs. Following 15 h incubation, the cells on the upper surface of the membranes were removed with cotton swabs, and the cells at the bottom of the filter were fixed with 100% ethanol for 10 min and stained with 0.1% crystal violet solution for 30 min. The dye was eluted using 33% acetic acid, and crystal violet absorbance was measured at 570 nm using the microplate reader.

### Cell damage measurement

Early cell damage was determined using the LDH cytotoxicity detection kit (Promega, Madison, WI), which quantifies the LDH release from the cells into the culture medium. The HCECs were seeded 5,000 cells per well in 96-well plates for 24 h to promote adherence. The cells were treated as the same as the evaluation of cell proliferation. Cell-free supernatants from the cultures were collected, passed through a 0.2-µm filter, and used in the LDH assay as instructed by the manufacturer. Maximum LDH release (high control) was determined by solubilizing cells with 1% Triton X-100, and spontaneous LDH release (low control) was determined by incubating cells with the medium alone. A reduction reaction of tetrazolium salt (INT) to a red formazan salt was used as an indicator of LDH activity in the supernatant. Absorbance was read at 490 nm by using a microplate reader. Results were quantified as (experimental value - low control/high control - low control) × 100.

### Cell viability detection

HCECs were seeded for 24 h and then incubated with different drugs for another 24 h. Next, the cells were collected by centrifugation and incubated by 7-AAD staining (1 µg/ml final concentration; 7-AAD; BD PharMingen, San Diego, CA). Cell mortality rate was measured using flow cytometry.

### Median lethal time assay

HCECs (3×10^5^ cells) were collected and treated with 100-μl undiluted anti-inflammatory eye drops for different exposure times. The treatment was stopped by 10-ml DMEM/F-12 media containing 10% FBS. The cells were then labeled by 7-AAD staining, and the cell viability was measured using flow cytometry. Median lethal time (LT_50_, time when the mortality rate was 50%) was counted according to the cell viability curve.

### Statistical analysis

Data are presented as mean±SD. The differences were analyzed by SPSS 10.0 (SPSS Inc., Chicago, IL) software (one-way ANOVA) with a significance value of p<0.05.

## Results

### Comparisons of corneal epithelial effects among anti-inflammatory eye drops

To evaluate the cellular effects of the four anti-inflammatory eye drops on HCECs, five assays were used in the present study. Acute cytotoxicity was evaluated by the measurement of median lethal time, chronic cellular effects were evaluated by the cell proliferation, migration, damage, and cell viability assay, in which corneal epithelial cells were treated with individual eye drops for 15 or 24 h.

**Figure 1:**
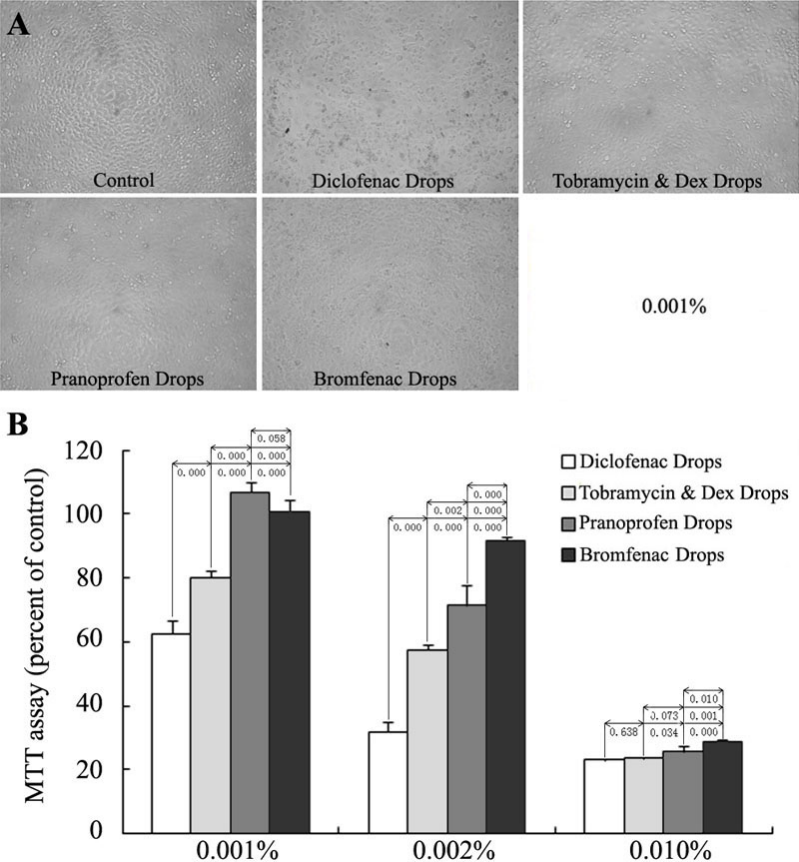
Effects of the four anti-inflammatory eye drops on the proliferation of cultured HCECs. HCECs were treated with 0.001%, 0.002%, and 0.010% eye drops for 24 h (n=4) and analyzed by morphological observation (**A**) and MTT assay (**B**). The inhibitory effects of the four eye drops were in a concentration-dependent manner, and the differences among the four solutions became less significant as the concentrations increased. All the experiment groups showed a significant inhibitory effect when compared with the control except for the Pranoprofen Eye Drops and Bromfenac Sodium Hydrate Eye Drops at the 0.001% concentration.

The effects on the proliferation of cultured HCECs were evaluated with MTT assay. HCECs were incubated with 0.001% (100 fold dilution), 0.002% (50 fold dilution), and 0.010% (10-fold dilution) eye drops for 24 h. The proliferation inhibition of the four eye drops was in a concentration-dependent manner, and the differences among the four solutions became less significant as the concentrations increased. All the experiment groups showed a significant inhibitory effect when compared with the control except for the Pranoprofen Eye Drops and Bromfenac Sodium Hydrate Eye Drops at the 0.001% concentration; there were no differences between these two groups (Figure 1A,B).

**Figure 2:**
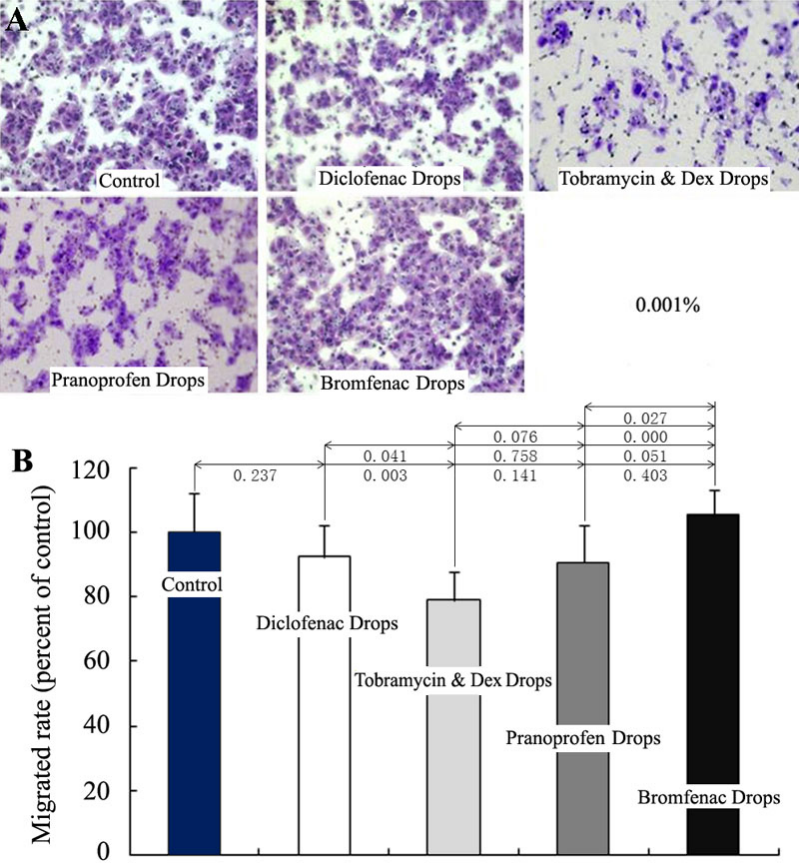
Influence of the four anti-inflammatory eye drops on the migration of cultured HCECs. HCECs were inoculated on the top of the transwell chamber and were induced to migrate toward the lower chambers containing the 0.001% eye drops and 1% FBS for 15 h (n=4). The transmigrated HCECs were stained with crystal violet and visualized with a microscope (**A**). The dye was eluted with acetic acid, and the crystal violet absorbance was measured (**B**). Only Tobramycin & Dex Eye Drops showed a significant difference compared with the control at the concentration.

Cell migration plays an important role in corneal wound healing. We used the transwell migration assay to quantify the effects of eye drops (0.001%) on the migration of cultured HCECs. The transmigrated cells were stained with crystal violet (Figure 2A) and the absorbance of elution was measured. There was no significant influence on HCEC migration compared with the control, except for Tobramycin & Dex Eye Drops (Figure 2B).

**Figure 3:**
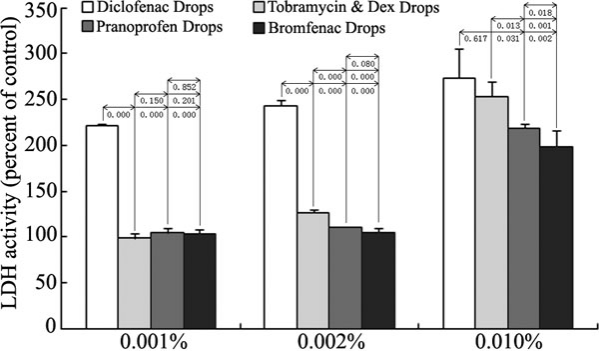
Effects of the four anti-inflammatory eye drops on cell damage of cultured HCECs. HCECs were treated with 0.001%, 0.002%, and 0.010% eye drops for 24 h (n=4). Cell supernatants were collected and detected by LDH assay. LDH release after exposure of cells to the eye drops was concentration dependent. Diclofenac Sodium Eye Drops showed the most significant toxicity among the four eye drops at the three concentration groups. At the higher 0.010% concentration, all four eye drops caused apparent cell damage and LDH releasing, and the differences among them became less significant.

**Figure 4:**
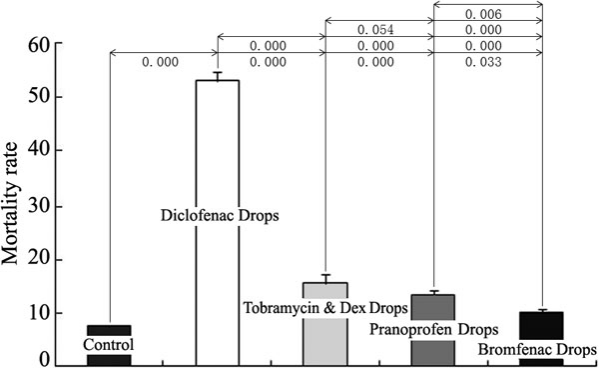
Effects of the four anti-inflammatory eye drops on the viability of cultured HCECs. HCECs were treated with 0.001% eye drops for 24 h (n=4). The cells were stained with 7-AAD, and cell mortality was analyzed using flow cytometry. All four eye drops showed significant effects on the viability of HCECs, and Diclofenac Sodium Eye Drops showed the most significant toxicity among the four eye drops.

Cellular toxicity always causes membrane damage and results in the releasing of LDH from the cytoplasma. By detecting the LDH in cell supernatant with 0.001%, 0.002%, and 0.010% eye drops, we found that Diclofenac Sodium Eye Drops showed the most significant toxicity among the four solutions at three concentration groups. At the higher 0.010% concentration, all four solutions caused apparent cell damage, LDH releasing, and the differences among them became less significant (Figure 3). The results were basically identical with the measurement of cell mortality by 7-AAD staining at the 0.001% concentration (Figure 4). The number of dead cells treated with Diclofenac Sodium Eye Drops showed almost 3–5 folds of the cells treated with the other three solutions.

**Figure 5:**
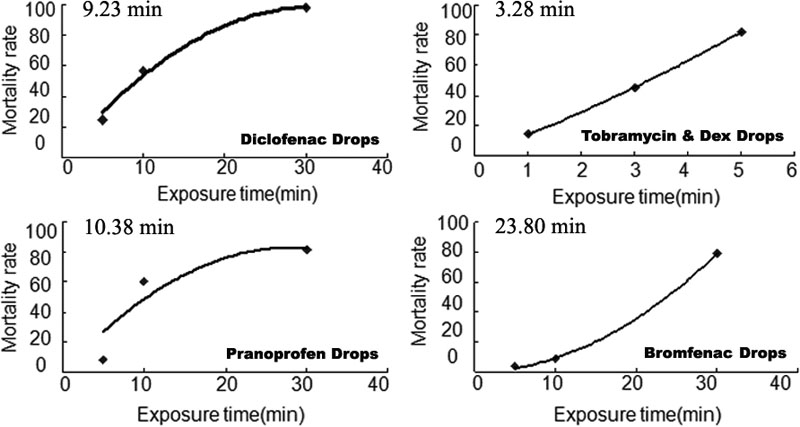
The LT50 of the four anti-inflammatory eye drops. HCECs were treated directly with 100-μl undiluted eye drops and stopped by 10-mL DMEM/F-12 media (n=4). The cells were labeled with 7-AAD staining, and the cell viability was measured using flow cytometry. The LT50 was counted according to the cell viability curve. The LT50 was 23.80 min for Bromfenac Sodium Hydrate Eye Drops, 10.38 min for Pranoprofen Eye Drops, 9.23 min for Diclofenac Sodium Eye Drops, and 3.28 min for Tobramycin & Dex Eye Drops.

Acute cytotoxicity was detected by treating the HCECs with the original eye drops. The LT_50_ of the four anti-inflammatory eye drops was 23.80 min for Bromfenac Sodium Hydrate Eye Drops, 10.38 min for Pranoprofen Eye Drops, 9.23 min for Diclofenac Sodium Eye Drops, and 3.28 min for Tobramycin & Dex Eye Drops (Figure 5), indicating that Tobramycin & Dex Eye Drops had the fastest toxicity effect, followed by Diclofenac Sodium Eye Drops, Pranoprofen Eye Drops, and Bromfenac Sodium Hydrate Eye Drops.

According to cell proliferation, damage, and cell viability assay, Bromfenac Sodium Hydrate Eye Drops had the least significant corneal epithelial effects whereas Diclofenac Sodium Eye Drops had the most significant corneal epithelial effects among the four anti-inflammatory eye drops. Tobramycin & Dex Eye Drops showed the most significant influence on cell migration and acute cytotoxicity.

### Comparison of corneal epithelial effects among the active components of anti-inflammatory eye drops

**Figure 6:**
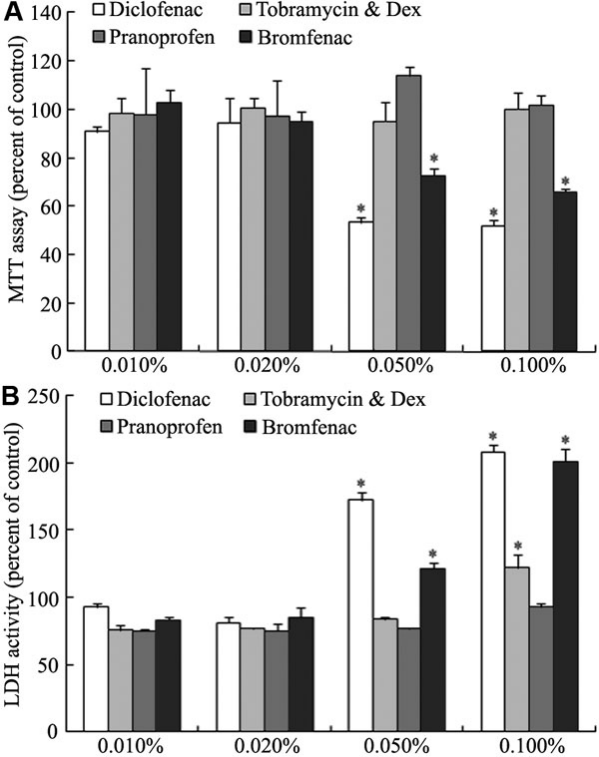
Effects of the active components of the four anti-inflammatory eye drops on cultured HCECs. HCECs were treated with 0.010%, 0.020%, 0.050%, and 0.100% solutions for 24 h (n=4) and analyzed by MTT assay (**A**) and LDH assay (**B**). Only diclofenac and bromfenac components showed significant cytotoxicity at the concentrations above 0.020% and 0.100%.

MTT and LDH assays were used to evaluate the cytotoxicity of the active components of the four anti-inflammatory eye drops. HCECs were incubated with 0.010%, 0.020%, 0.050%, and 0.100% (the same concentrations as the original eye drops) active component solutions for 24 h. The corneal epithelial toxicity differences among the active components of the four eye drops became significant as the concentration increased above 0.020%. Only diclofenac and bromfenac components showed significant cytotoxicity at the higher concentrations (0.050% and 0.100%; Figure 6).

### Comparison of corneal epithelial effects between anti-inflammatory eye drops and their active components

**Figure 7:**
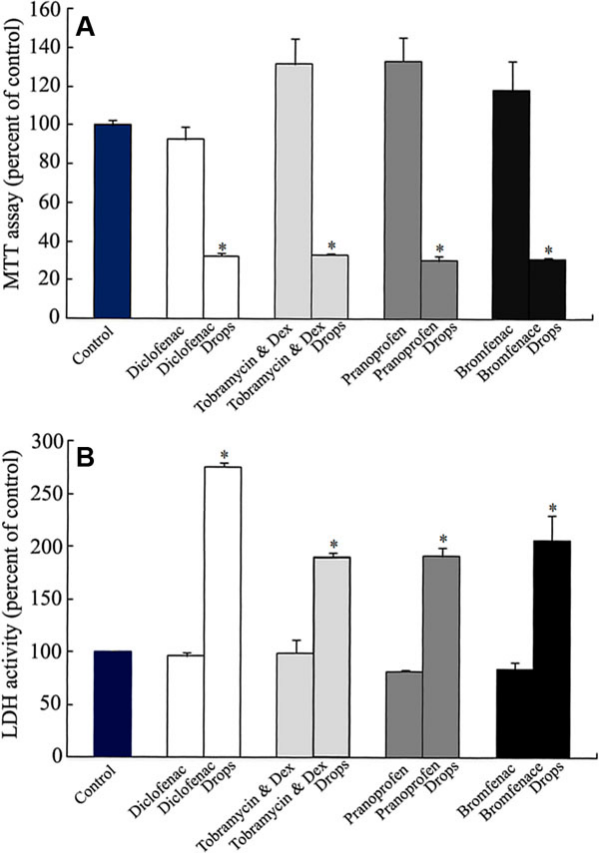
Comparisons of corneal epithelial toxicity of anti-inflammatory eye drops and their active components. HCECs were treated with either 0.010% eye drops or 0.010% active components for 24 h (n=4). The corneal epithelial toxicity was measured by MTT assay (**A**) and LDH assay (**B**). Significant differences were between the eye drops and the corresponding active components.

To confirm the cytotoxic origin, MTT and LDH assays were used to evaluate the differences between the four anti-inflammatory eye drops and their corresponding active components at the 0.010% concentration (Figure 7). The active component groups showed no cytotoxicity toward HCECs compared with the control group. The four anti-inflammatory eye drops showed significant toxicity on the proliferation and damage of HCECs when compared with the control and the active components. The cellular toxicity of the four anti-inflammatory eye drops did not originate from the active components but from the preservatives or other ingredients.

## Discussion

Ocular epithelial disorders after eye surgery can result in visual deterioration and patient discomfort, which may be caused by drug toxicity. In the present study, we systematically evaluated the overall cellular effects of four commercially available anti-inflammatory eye drops and their active components on HCECs in vitro. The results indicated that Diclofenac Sodium Eye Drops (contains 0.1% diclofenac sodium) showed significant effects on cell damage and viability, which was consistent with previous reports [7,11,12]. The cytotoxicity of diclofenac was represented by retarding corneal epithelial healing [8,13], decreasing cell growth [14], and causing the higher incidence of persistent epithelial defects [15]. The mechanism related with diclofenac not only blocks the COX pathway but also diminishes the pool of available arachidonic acid. Arachidonic acid is an important component of cellular structural integrity; the lack of its availability and the potentially increased permeability of cellular membranes may lead to corneal epithelial cell death and keratolysis [16,17]. It should be mentioned that Tobramycin & Dex Eye Drops inhibited the migration and showed the quickest effect on cell viability in the study. However, the inhibitory effect on corneal epithelial migration was related with Dex, and the inhibition of cell viability was caused by the presence of Tobramycin, according to our previous study (data not shown).

It should be mentioned that there were significant differences between acute and chronic cellular effects among these four anti-inflammatory eye drops. As previous description, steroid levels in the cornea decrease by 50% within 1 h after topical application [18], while the aqueous humor concentrations of topically applied Diclofenac and Bromfenac gradually decreased until complete removal after approximately 24 h because of the rapid turnover of tear, aqueous humor, etc. [19,20]. However, the concentrations of eye drops exposed to human corneal epithelial cells in vitro remained constant, which represents the early higher concentrations when topical application in vivo. For these reasons, the 24 h exposure of cultured epithelial cells to eye drops in the present study was parallel with the long-term exposure time and concentration experienced by epithelial cells in vivo. Moreover, the HCECs showed significant toxicity when exposed to the constant concentrations of eye drops in vitro, so we measured the acute cellular toxicity within 1 h, while measured the chronic cellular toxicity within 24 h. More importantly, we first found significant differences between the eye drops and their active components under the 0.010% concentration, but we did not find any differences among their active components at the same concentrations. Moreover, the pH values of 0.001%, 0.002%, and 0.010% eye drops diluted in cell culture media became almost identical (data not shown). The above results suggest that the corneal epithelial toxicity of the four anti-inflammatory eye drops were independent in their active components or pH values, especially in the lower concentrations (<0.010%).

As reported previously, preservatives and buffering agents are essential ingredients except of active components in eye drops. They provide a level of antimicrobial activity and prolong the half-life of the drug by preventing biodegradation and maintaining drug potency [21]. However, long-term topical use may cause tear film instability, loss of goblet cells, ocular epithelial squamous metaplasia and apoptosis, disruption of corneal epithelium barrier, and damage to deeper ocular tissues. Therefore, care should be taken to avoid long-term use of preservative-containing eye drops or to use preservative-free solutions whenever possible. Diclofenac sodium eye drops containing thimerosal from Japan or Europe caused punctuating corneal epithelial damage, whereas the USA products containing sorbic acid or the diclofenac sodium solution alone had no significant toxicity. Because the present study had limited product information about the components, contents, and possible interactions between the preservatives and other agents, it is necessary to further investigate the origin of cellular toxicity.
